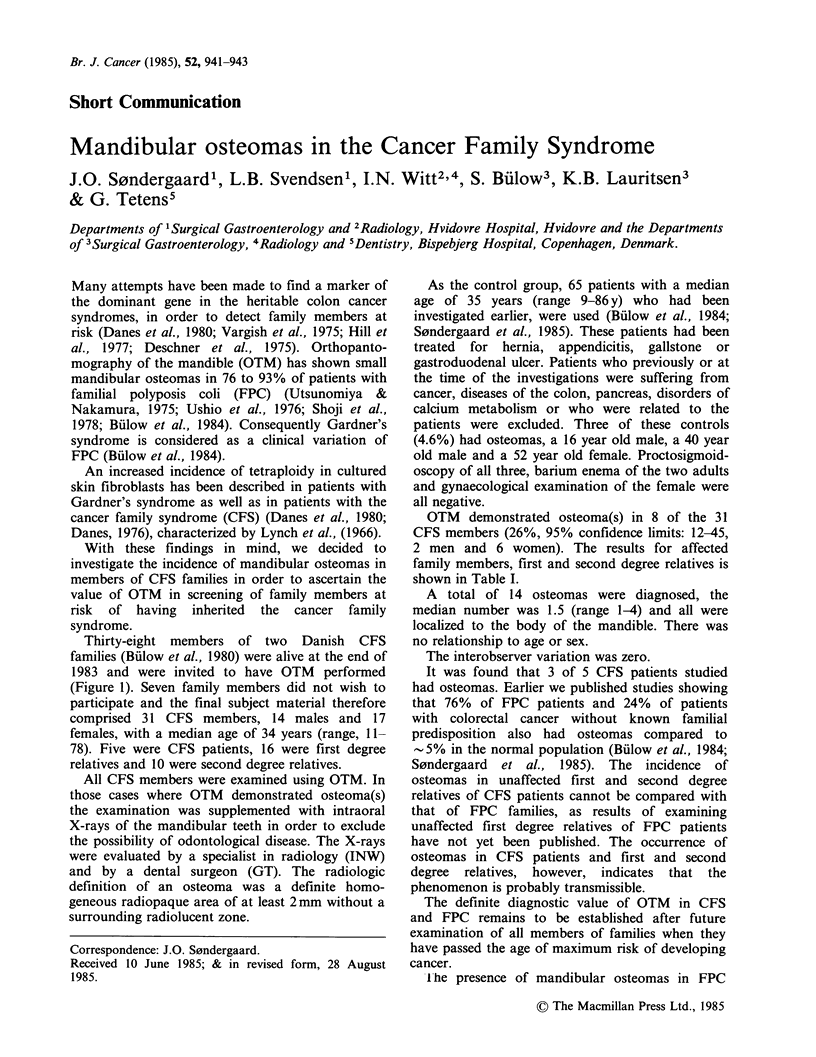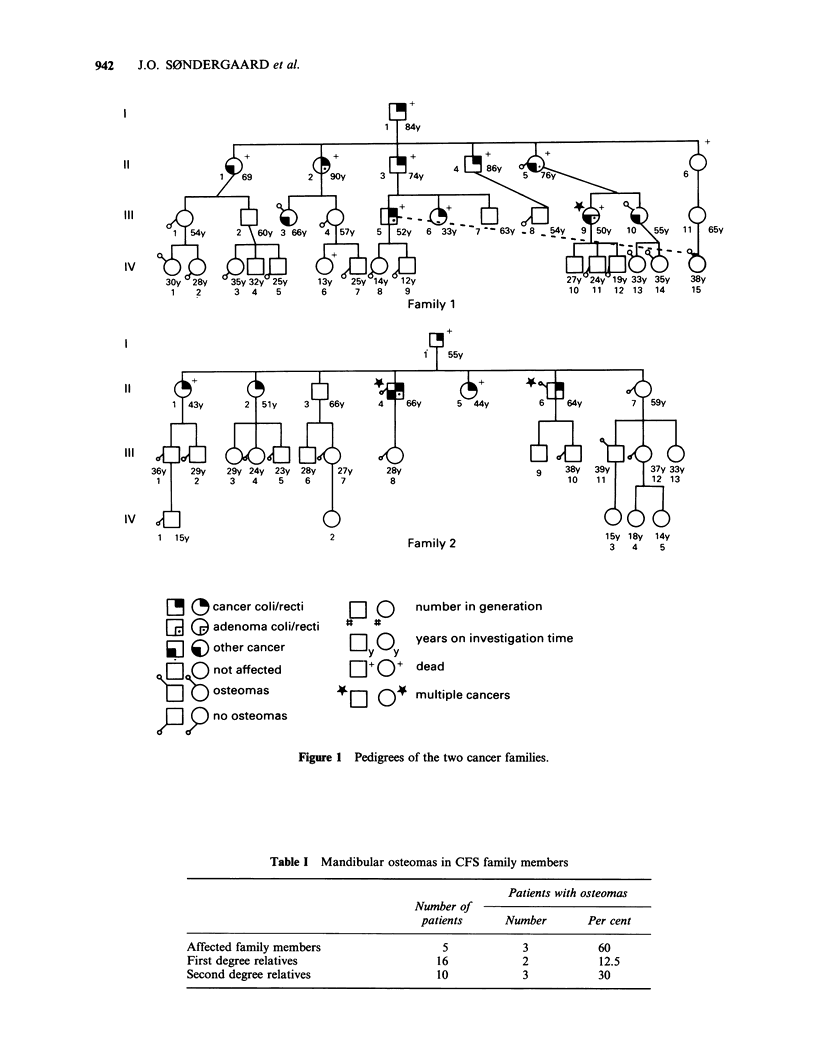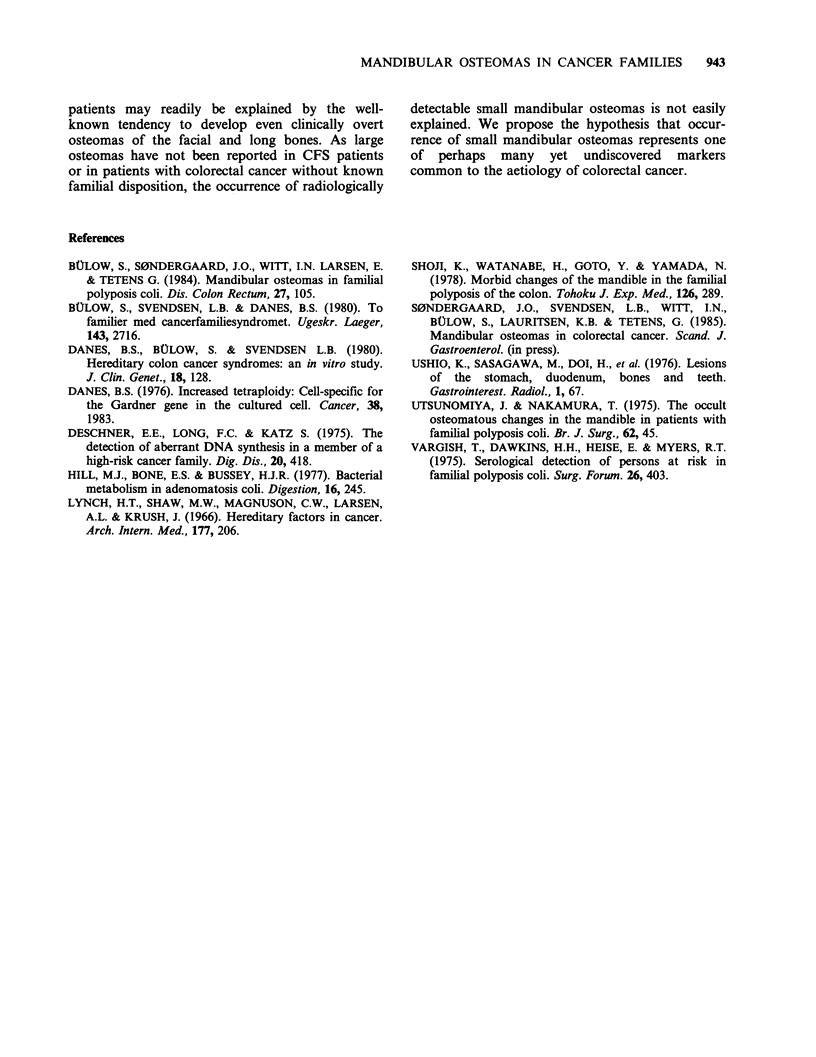# Mandibular osteomas in the cancer family syndrome.

**DOI:** 10.1038/bjc.1985.281

**Published:** 1985-12

**Authors:** J. O. Søndergaard, L. B. Svendsen, I. N. Witt, S. Bülow, K. B. Lauritsen, G. Tetens


*Br. J. Cancer* (1985), **52**, 941–943

### Short Communication

### Mandibular osteomas in the Cancer Family Syndrome

J.O. Søndergaard[1], L.B. Svendsen[1], I.N. Witt[2,4], S. Bülow[3], K.B. Lauritsen[3] & G. Tetens[5]

*Departments of [1]Surgical Gastroenterology and [2]Radiology, Hvidovre Hospital, Hvidovre and the Departments of [3]Surgical Gastroenterology, [4]Radiology and [5]Dentistry, Bispebjerg Hospital, Copenhagen, Denmark.*

Many attempts have been made to find a marker of the dominant gene in the heritable colon cancer syndromes, in order to detect family members at risk (Danes *et al.*, 1980; Vargish *et al.*, 1975; Hill *et al.*, 1977; Deschner *et al.*, 1975). Orthopantomography of the mandible (OTM) has shown small mandibular osteomas in 76 to 93% of patients with familial polyposis coli (FPC) (Utsunomiya & Nakamura, 1975; Ushio *et al.*, 1976; Shoji *et al.*, 1978; Bülow *et al.*, 1984). Consequently Gardner's syndrome is considered as a clinical variation of FPC (Bülow *et al.*, 1984).

An increased incidence of tetraploidy in cultured skin fibroblasts has been described in patients with Gardner's syndrome as well as in patients with the cancer family syndrome (CFS) (Danes *et al.*, 1980; Danes, 1976), characterized by Lynch *et al.*, (1966).

With these findings in mind, we decided to investigate the incidence of mandibular osteomas in members of CFS families in order to ascertain the value of OTM in screening of family members at risk of having inherited the cancer family syndrome.

Thirty-eight members of two Danish CFS families (Bülow *et al.*, 1980) were alive at the end of 1983 and were invited to have OTM performed (Figure 1). Seven family members did not wish to participate and the final subject material therefore comprised 31 CFS members, 14 males and 17 females, with a median age of 34 years (range, 11–78). Five were CFS patients, 16 were first degree relatives and 10 were second degree relatives.

All CFS members were examined using OTM. In those cases where OTM demonstrated osteoma(s) the examination was supplemented with intraoral X-rays of the mandibular teeth in order to exclude the possibility of odontological disease. The X-rays were evaluated by a specialist in radiology (INW) and by a dental surgeon (GT). The radiologic definition of an osteoma was a definite homogeneous radiopaque area of at least 2 mm without a surrounding radiolucent zone.

Correspondence: J.O. Søndergaard.
Received 10 June 1985; & in revised form, 28 August 1985.

As the control group, 65 patients with a median age of 35 years (range 9–86 y) who had been investigated earlier, were used (Bülow *et al.*, 1984; Søndergaard *et al.*, 1985). These patients had been treated for hernia, appendicitis, gallstone or gastroduodenal ulcer. Patients who previously or at the time of the investigations were suffering from cancer, diseases of the colon, pancreas, disorders of calcium metabolism or who were related to the patients were excluded. Three of these controls (4.6%) had osteomas, a 16 year old male, a 40 year old male and a 52 year old female. Proctosigmoidoscopy of all three, barium enema of the two adults and gynaecological examination of the female were all negative.

OTM demonstrated osteoma(s) in 8 of the 31 CFS members (26%, 95% confidence limits: 12–45, 2 men and 6 women). The results for affected family members, first and second degree relatives is shown in Table I.

A total of 14 osteomas were diagnosed, the median number was 1.5 (range 1–4) and all were localized to the body of the mandible. There was no relationship to age or sex.

The interobserver variation was zero.

It was found that 3 of 5 CFS patients studied had osteomas. Earlier we published studies showing that 76% of FPC patients and 24% of patients with colorectal cancer without known familial predisposition also had osteomas compared to ~5% in the normal population (Bülow *et al.*, 1984; Søndergaard *et al.*, 1985). The incidence of osteomas in unaffected first and second degree relatives of CFS patients cannot be compared with that of FPC families, as results of examining unaffected first degree relatives of FPC patients have not yet been published. The occurrence of osteomas in CFS patients and first and second degree relatives, however, indicates that the phenomenon is probably transmissible.

The definite diagnostic value of OTM in CFS and FPC remains to be established after future examination of all members of families when they have passed the age of maximum risk of developing cancer.

The presence of mandibular osteomas in FPC

© The Macmillan Press Ltd., 1985

**Figure 1** Pedigrees of the two cancer families.

**Table I** Mandibular osteomas in CFS family members

| | Number of patients | Patients with osteomas | |
|---|---|---|---|
| | | *Number* | *Per cent* |
| Affected family members | 5 | 3 | 60 |
| First degree relatives | 16 | 2 | 12.5 |
| Second degree relatives | 10 | 3 | 30 |

patients may readily be explained by the well-known tendency to develop even clinically overt osteomas of the facial and long bones. As large osteomas have not been reported in CFS patients or in patients with colorectal cancer without known familial disposition, the occurrence of radiologically detectable small mandibular osteomas is not easily explained. We propose the hypothesis that occurrence of small mandibular osteomas represents one of perhaps many yet undiscovered markers common to the aetiology of colorectal cancer.

**References**

BÜLOW, S., SØNDERGAARD, J.O., WITT, I.N. LARSEN, E. & TETENS G. (1984). Mandibular osteomas in familial polyposis coli. *Dis. Colon Rectum,* **27,** 105.

BÜLOW, S., SVENDSEN, L.B. & DANES, B.S. (1980). To familier med cancerfamiliesyndromet. *Ugeskr. Laeger,* **143,** 2716.

DANES, B.S., BÜLOW, S. & SVENDSEN L.B. (1980). Hereditary colon cancer syndromes: an *in vitro* study. *J. Clin. Genet.,* **18,** 128.

DANES, B.S. (1976). Increased tetraploidy: Cell-specific for the Gardner gene in the cultured cell. *Cancer,* **38,** 1983.

DESCHNER, E.E., LONG, F.C. & KATZ S. (1975). The detection of aberrant DNA synthesis in a member of a high-risk cancer family. *Dig. Dis.,* **20,** 418.

HILL, M.J., BONE, E.S. & BUSSEY, H.J.R. (1977). Bacterial metabolism in adenomatosis coli. *Digestion,* **16,** 245.

LYNCH, H.T., SHAW, M.W., MAGNUSON, C.W., LARSEN, A.L. & KRUSH, J. (1966). Hereditary factors in cancer. *Arch. Intern. Med.,* **177,** 206.

SHOJI, K., WATANABE, H., GOTO, Y. & YAMADA, N. (1978). Morbid changes of the mandible in the familial polyposis of the colon. *Tohoku J. Exp. Med.,* **126,** 289.

SØNDERGAARD, J.O., SVENDSEN, L.B., WITT, I.N., BÜLOW, S., LAURITSEN, K.B. & TETENS, G. (1985). Mandibular osteomas in colorectal cancer. *Scand. J. Gastroenterol.* (in press).

USHIO, K., SASAGAWA, M., DOI, H., *et al.* (1976). Lesions of the stomach, duodenum, bones and teeth. *Gastrointerest. Radiol.,* **1,** 67.

UTSUNOMIYA, J. & NAKAMURA, T. (1975). The occult osteomatous changes in the mandible in patients with familial polyposis coli. *Br. J. Surg.,* **62,** 45.

VARGISH, T., DAWKINS, H.H., HEISE, E. & MYERS, R.T. (1975). Serological detection of persons at risk in familial polyposis coli. *Surg. Forum.* **26,** 403.